# Emerging Roles of Estrogen-Regulated Enhancer and Long Non-Coding RNAs

**DOI:** 10.3390/ijms21103711

**Published:** 2020-05-25

**Authors:** Melina J. Sedano, Alana L. Harrison, Mina Zilaie, Chandrima Das, Ramesh Choudhari, Enrique Ramos, Shrikanth S. Gadad

**Affiliations:** 1 Center of Emphasis in Cancer, Department of Molecular and Translational Medicine, Paul L. Foster School of Medicine, Texas Tech University Health Sciences Center El Paso, El Paso, TX 79905, USA; melina.j.sedano@ttuhsc.edu (M.J.S.); Alana.L.Harrison@ttuhsc.edu (A.L.H.); ramesh.choudhari@ttuhsc.edu (R.C.); enrique.ramos@ttuhsc.edu (E.R.); 2 Department of Molecular and Translational Medicine, Paul L. Foster School of Medicine, Texas Tech University Health Sciences Center El Paso, El Paso, TX 79905, USA; 3 Graduate School of Biomedical Sciences, Texas Tech University Health Sciences Center El Paso, El Paso, TX 79905, USA; Mina.Zilaie@ttuhsc.edu; 4 Biophysics and Structural Genomics Division, Saha Institute of Nuclear Physics, 1/AF Bidhannagar, Kolkata 700064, India; chandrima.das@saha.ac.in; 5 Homi Bhaba National Institute, Mumbai 400094, India

**Keywords:** estrogen, eRNA, lncRNA, breast cancer, transcription, gene-regulation, estrogen receptor

## Abstract

Genome-wide RNA sequencing has shown that only a small fraction of the human genome is transcribed into protein-coding mRNAs. While once thought to be “junk” DNA, recent findings indicate that the rest of the genome encodes many types of non-coding RNA molecules with a myriad of functions still being determined. Among the non-coding RNAs, long non-coding RNAs (lncRNA) and enhancer RNAs (eRNA) are found to be most copious. While their exact biological functions and mechanisms of action are currently unknown, technologies such as next-generation RNA sequencing (RNA-seq) and global nuclear run-on sequencing (GRO-seq) have begun deciphering their expression patterns and biological significance. In addition to their identification, it has been shown that the expression of long non-coding RNAs and enhancer RNAs can vary due to spatial, temporal, developmental, or hormonal variations. In this review, we explore newly reported information on estrogen-regulated eRNAs and lncRNAs and their associated biological functions to help outline their markedly prominent roles in estrogen-dependent signaling.

## 1. Introduction

Technological advancements in molecular biology have improved our ability to identify the novel coding and non-coding genes that make up the human genome. Information extracted from high throughput sequencing data has facilitated the identification of disease-causing genes leading to new treatments, and even cures to many heritable diseases [1,2]. Within the transcribed genome, there are three classes of RNAs that have been extensively analyzed, which include messenger RNA (mRNA), ribosomal RNA (rRNA), and transfer RNA (tRNA) [3]. The non-coding genome consists of different sizes and types of RNAs, many of which were initially considered to be merely background noise. That theory has been thoroughly debunked, and in fact, non-coding RNAs (ncRNAs) have been found to modify transcriptional processes in conjunction with environmental and physiological changes [4]. The transcriptional changes caused by non-coding genes have often been linked to many cancers and other disorders [1,2]. Some of the most outstanding scientific advancements used to identify ncRNA include RNA-seq, which yields the transcriptome profile and quantifies the different isoforms and transcript levels [5], Global nuclear Run-On Sequencing (GRO-seq), and Precision nuclear Run-on Sequencing (PRO-seq), which function to capture primary and mature transcripts, respectively [6]. In-situ hybridization studies using labeled RNA probes can help not only to visualize but also to locate other neighboring transcripts involved in the biological process [7]. Furthermore, chromatin immunoprecipitation assays coupled with deep-sequencing (ChIP-seq), evaluate DNA-protein interactions, which can be used to identify transcription factors regulating non-coding genes [8]. ncRNAs are technically divided into two categories based on their size—one group includes ncRNAs that are less than 200nt long, and the other group is composed of ncRNAs that are more than 200nt long, deemed long non-coding RNAs (lncRNAs) [1,2]. In contrast, enhancer RNAs (eRNAs) have been found to range in size [6]. ncRNAs are also classified based on a number of distinguishing factors such as essential functions, location, direction of transcription, tissue location, developmental roles, or response to stimuli [1,2]. LncRNAs and eRNAs play a prominent role in estrogen-dependent biological processes, and their role in cancer development and progression has become an area of interest due to their potential as future biomarkers and treatment targets [6,9]. In this review, we summarize new information on estrogen-regulated lncRNAs and eRNAs to help highlight their roles in estrogen-dependent cellular processes.

## 2. Estrogen Signaling

**Figure 1:**
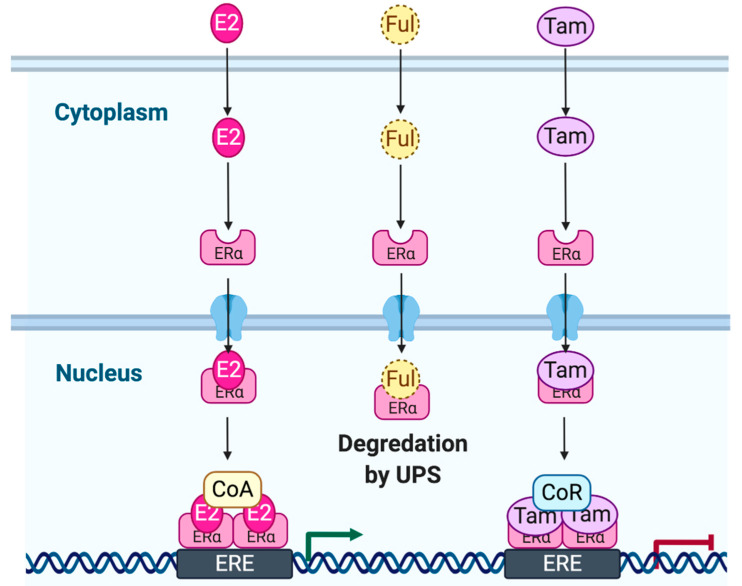
Simplistic representation of estrogen receptor (ER) signaling (genomic activity). Estrogen (E2)-bound ERα, is recruited to target promoters at estrogen response elements (EREs). E2 binds ERα and recruits coactivator complexes (CoA) to modulate gene transcription. Tamoxifen (TAM) and fulvestrant (Ful) are E2 antagonists. UPS: ubiquitin–proteasome system; CoR: Coregulator.

Endocrine signaling operates through the availability of nuclear hormone receptors, which act as members of the steroid/thyroid hormone receptor superfamily, a group also including structurally related receptors for androgens, thyroid hormones, progesterone, mineralocorticoids, vitamin D, retinoic acid, and estrogen, among others. There are two estrogen receptors (ERs), ERα and ERβ, and while both receptor effects are almost always regulated by the steroid hormone estradiol (E2), exclusive cell-type-specific expression in both reproductive and non-reproductive tissues are observed [10]. ERs have well-defined structural components, which include a DNA-binding domain (DBD), a nuclear localization signal (NLS), and a ligand-binding domain (LBD) [10]. The LBD is where the hormone-binding site, dimer joining site, and ligand-dependent co-regulator interaction site are located [11,12]. The ligand-dependent co-regulator pocket-size differs between ERα and ERβ, indicating ER subtype-specific ligand binding, accounting for the site-specific mechanisms of the two subtypes [10]. The general mechanism of estrogen signaling initiates with the release of E2 (17β-estradiol) into the cells. ER protein, which is generally localized in the peri-membrane and nucleus of cells [13], is modulated by multiple transcription factors at each location in a time and location-restricted manner (Figure 1). The membrane-associated ER signaling pathway (G protein-coupled estrogen receptor) is part of the array of signaling cascades affecting breast cancer cell proliferation and migration [14]. In addition to their presence in the peri-membrane and nucleus, studies have confirmed additional ER accumulation in the mitochondria; furthermore, E2 has been found to target mitochondria in reproductive cell types, in both a cell and ER subtype/isoform-dependent manner [15]. While the exact mechanisms have not been fully described, an E2-activated mitochondrial ER mechanism could be responsible for any number of signal cascades, of which tumor cell survival is an end result [15,16]. Within minutes of E2 exposure, E2 binds to the ERα receptor, which dimerizes and binds to the genome either directly via ERα binding sites (ERBs) (Figure 1), or indirectly through transcription factors such as NF-κB and CREB, which link ER to co-activators or co-repressors [17,18,19]. Multiple modifications, such as phosphorylation, glycosylation, and acetylation, among others, are necessary for ER-dependent signaling [20]. After binding, the signaling cascade induces the recruitment of coregulator proteins/complexes, which may interact directly or indirectly through scaffolding coregulators. The coregulators can induce chromatin remodeling [21], chromatin looping [22], or post-translational modification of transcription-related proteins [23]. Once the looping events bring together enhancers with target promoters, RNA polymerase II is activated. Non-coding RNAs have so far been found to play a role in coregulator interactions to enhance ERα-dependent transcriptional changes, histone acetylation, and enhancer-promoter looping interactions. The role of these non-coding RNAs will be discussed in this review. 

## 3. Enhancer RNAs (eRNAs)

**Figure 2:**
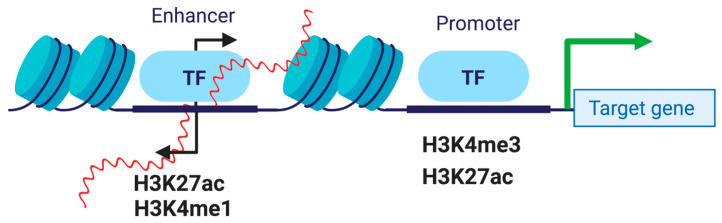
eRNA transcription at enhancers. Pol II transcribes eRNAs from active enhancers marked with H3K4me1 and H3K27ac.

Enhancer RNAs are transcribed bidirectionally by Polymerase II from transcriptionally active enhancer regions marked by H3K27ac and H3K4me1 (Figure 2). Enhancer RNAs have been found to increase the expression of target genes when stimulated and have also been associated with stabilizing the binding of activating transcription factors, regardless of orientation or promoter location [24]. The target loci are stimulated during specific developmental stages in different but select tissue, and cell types, meaning transcription for active enhancers happens only in a spatially and temporally restricted manner [25]. 

**Figure 3:**
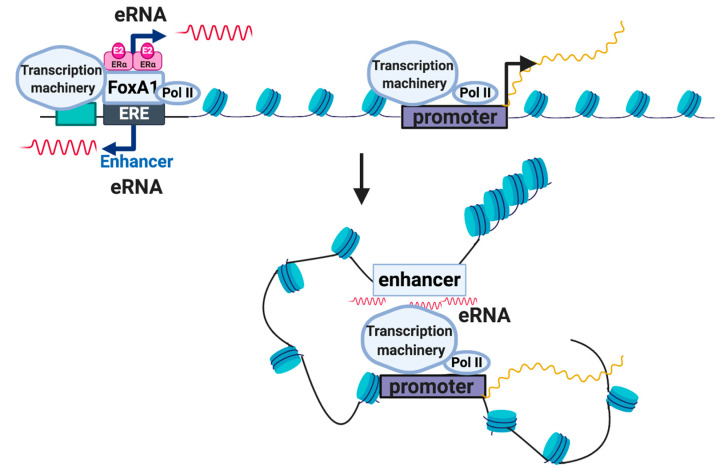
A plausible mechanism of estrogen-induced enhancer RNA (eRNA) function in chromatin looping.

Other recently annotated distinguishing characteristics of eRNAs include undetectable levels of H3K4me3, lower expression levels compared to lncRNAs, a shorter length, and limited signs of splicing or polyadenylation [26]. Even so, there have been ncRNAs found to be larger, spliced, polyadenylated, and with histone modification patterns similar to lncRNAs, yet have still been defined as an eRNA based on the deletion of its genomic region/terminating transcription and its effect on gene expression [27]. Previous studies on estrogen receptor α (ERα) and estrogen binding sites (ERBS) in breast cancer cell lines have shown a particular class of eRNAs that are mostly upregulated by estrogen; furthermore, features such as open chromatin, and enrichment of H3K4me1, p300/CBP, and RNA polymerase II are enhanced when estrogen-associated eRNA production is promoted [6,28]. While either estrogen or androgens can induce enhancer response, exposure to such stimuli can lead to different eRNA expression in different tissues, due to eRNA tissue specificity [29]. As discussed by Li W et al., as soon as ERα is bound by estrogen, the body responds by increasing eRNA transcription on enhancers next to estrogen-upregulated coding genes (Figure 3) [29]. They found that the induced eRNAs have a stabilizing role in ligand-dependent induction of estrogen-dependent coding, as well as non-coding genes. Knockdown analysis of specific eRNAs led to the reduction of a protein complex that regulates the separation of sister chromatids, known as cohesin. Cohesin is believed to contribute to the stabilization of looping formed by estrogen, ERα, and the enhancer RNA promoter, which is required to activate genes [29]. Estrogen-regulated eRNAs are still being identified; among those found to be part of the enhancer-promoter looping stabilizers are *TFF1e*, *FOXC1e*, and *CA12e* (Table 1). *TFF1e* is an intronic eRNA located at the *TMPRSS3* gene at transmembrane protease serine 3, which is a pro-metastatic mediator found to have higher expression in breast, pancreatic, and colorectal cancers [30]. Based on bioinformatic analysis of possible binding proteins at *TMPRSS3* and due to its high expression, *TMPRSS3* is considered a key regulator of cancer pathways [30]. In addition to stabilizing specific enhancer-promoter looping mechanisms, eRNAs can also control ligand-dependent target gene transcription, and enhancer activity [31]. By characterizing these eRNAs and linking them to specific cancers, diagnostic and prognostic evaluations could significantly improve treatment and survival outcomes.

## 4. Estrogen-Regulated Long Non-Coding RNAs (lncRNAs)

**Figure 4:**
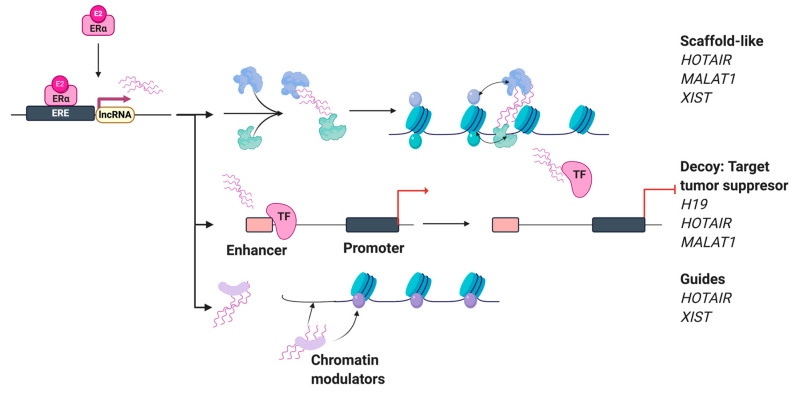
Known long non-coding RNA (lncRNA) mechanisms of action in regulating estrogen-dependent transcription. (TF: transcription factor; ERα estrogen receptor alpha; ERE: estrogen response element).

The potential function of lncRNAs in transcriptional regulation has sparked several investigations [1,2]. Previously, lncRNAs were thought to be transcriptional noise; however, they have emerged as critical regulators of biological functions. Regardless, for many lncRNAs, their exact functions still remain unclear to this day [32]. LncRNAs are a class of non-coding RNAs that are longer than 200 nucleotides long and are similar to mRNAs but have limited coding potential. The term lncRNA categorizes a group of ncRNA, which could include antisense RNAs, transcribed ultra-conserved regions (T-UCRs), divergent transcripts, intronic lncRNAs, and intergenic lncRNAs. So far, the functional roles of identified lncRNAs are vast and include regulating mRNA splicing, acting as miRNA loss of function tools, guides, decoys, scaffolds for chromatin modeling complexes, as well as controling post-transcriptional mRNA decay, etc. (Figure 4) [33]. The most common function of lncRNAs relates to epigenetic regulation, including transcriptional repression [34]. Novel cancer-related lncRNAs have been identified by comparing paired normal to cancerous tissue or cell type data from transcriptome sequencing (RNA-seq). Advances in next-generation sequencing techniques have expedited the discovery and annotation of lncRNAs in breast cancer [35] and other tissues [36]. These lncRNAs have been identified via GRO- and RNA-seq techniques, and many of them are regulated by estrogen, appearing to have specific roles in estrogen-dependent transcriptional regulation and cell cycle proliferation of ERα-positive MCF-7 human breast cancer cells [35]. When relating to tumorigenesis, lncRNAs affect splicing, the stability of the mRNA/protein interaction, and the specific site protein is found within the cell [4]. New information on the potential role of estrogen-regulated lncRNAs in human cancers can elucidate potential clinical applications and highlight exploratory objectives for future direction. Specific estrogen-regulated lncRNAs such as long non-coding RNA 152 (*LncRNA152*) and long non-coding RNA 67 (*LncRNA67*) show significantly higher relative expression levels in ER-positive (ER+) breast tumors in comparison to non-cancerous breast tissue, and upon estrogen treatment, can regulate cell proliferation and mitogenic division in ER+ cell lines [35]. This was further confirmed by the siRNA-mediated knockdown of *lncRNA152* and *lncRNA67* in MCF-7 cells, which showed a reduction in cell proliferation and changes in expression of cell cycle genes but not in non-ER- or low ER-expressing cell lines (MDAMB231; ER-negative breast cancer cell line) [35]. Further, siRNA-mediated knockdown could be rescued by Dox-induced ectopic expression of *lncRNA152* and *lncRNA67* [35]. Specifically, *lncRNA152* and *lncRNA67* appear to be involved in estrogen-dependent as well as estrogen-independent proliferation. 

Interestingly, an imprinting gene, known as H19 imprinted maternally expressed transcript (*H19*), produces a lncRNA expressed in a variety of cancer cells that works through different mechanisms to affect the progression and development of each cancer [37]. Further analysis showed that the knockdown of H19 led to increased DNA Methyltransferase 3 Beta (DNMT3B)-mediated methylation of a lncRNA encoding gene (Nctc1), within Igf2-H19-Nctc1. In vivo and in vitro studies showed the effect of an antisense lncRNA H19 could increase tumorigenic properties to ER-negative type cell line (MDAMB231) “An antisense lncRNA (91H) to *H19* was found to regulate *H19* expression levels via epigenetic modification and increasing tumorigenic properties of MDAMB231 cells [38].” Like several lncRNAs (Table 2), the *H19* lncRNA is also estrogen-regulated and has been found to affect cell proliferation and differentiation. The molecular roles associated with this lncRNA include aberrant DNA methylation, microRNA encoding, and a competing endogenous RNA (ceRNA) regulatory network [38]. Since it has also been shown to be an oncogene regulating malignant breast cell proliferation, it can be used as a biomarker in tracking breast cancer progression [38]. In the case of endocrine therapy-resistant cancer, *H19* expression is linked to increased ERα expression; therefore, downregulating *H19* can provide an alternative treatment for therapy-resistant cells with ER+ breast cancer [39,40]. These studies open up new opportunities to understand and identify novel molecules critical for estrogen-regulated pathways. Here, in this review, we summarize the role of key lncRNAs in estrogen-dependent signaling.

### 4.1. HOX Transcript Antisense RNA (HOTAIR) and Metastasis-Associated Lung Adenocarcinoma Transcript 1 (MALAT1)

The HOX loci are known for the expression of many lncRNAs in normal breast epithelia, in the primary tumor, and in metastases. *HOTAIR* lncRNA, from the mammalian HOX loci, is a potential clinical diagnostic marker for breast cancer since its expression profile appears to be increased in human primary breast tumors and distant metastases [41]. *HOTAIR* is transcribed in an antisense direction from the HOX C locus and is regulated in an estrogen-dependent manner via binding of ERα and coregulators to the numerous functional EREs on the *HOTAIR* promoter [51]. *HOTAIR* targets histone H3 lysine 27 trimethylation (H3K27me3) via the polycomb repressive complex 2 (PRC2), modulating metastasis-associated genes by defining chromatin states and their anatomic demarcation on fibroblasts [41,52]. In cancer, *HOTAIR* reverses the chromatin states, allowing changes in the gene expression to affect cell motility and matrix invasion. Further, Gupta et al. found that knockdown of *HOTAIR* in MCF-7 cell lines diminishes cell-matrix invasiveness in Matrigel-based assays, which could indicate a possible therapeutic target. Besides, silencing *HOTAIR* in doxorubicin-resistant cells decreases cell proliferation and increases apoptosis, which could benefit doxorubicin-resistant therapy [53]. 

Similarly to *HOTAIR*, Aiello et al. found that lncRNA *MALAT1* is regulated by estrogen and associates with estrogen response elements (EREs) and the polycomb repressive complex I (PRC1). The association of this lncRNA with PRC1, which is associated with growth control genes in the nucleus, indicates it localizes to chromatin [46,47]. Both in vivo and ex vivo experiments assessed by ChIP, RNA-chromatin immunoprecipitation (RIP), and chromatin isolation by RNA purification (ChIRP) assays, showed that *MALAT1* binding to chromatin and other components is attenuated when estrogen is present, suggesting it has an inhibitory role in regulating estrogen target genes in basal conditions [54]. Overall, these studies indicate that *HOTAIR* and *MALAT1* are involved in regulating estrogen-dependent functions in normal as well as in disease conditions, which can be explored as diagnostic and therapeutic targets.

### 4.2. Myocardial Infarction-Associated Transcript (MIAT)

The lncRNA *MIAT* was found to be originally associated with myocardial infarction [55] but has since become associated with other diverse conditions, such as diabetic retinopathy [56] and paranoid schizophrenia [57]. It was also found to have higher expression in ER+ breast cancer tissues and is highly expressed in high-grade ductal breast tumor tissues [42]. In ER+ breast cancer cells, *MIAT* is induced by estrogen and controlled by ERα. It was found that siRNA-mediated knockdown of *MIAT* inhibited cell cycle progression, whereas its overexpression increased cell proliferation in MCF-7 cells, with its mechanistic action being revealed as a regulator of the cell cycle transition from G1 to S phase [42].

When *MIAT* is downregulated, cancer cell proliferation is repressed, cellular apoptosis is induced, and cell senescence occurs, making *MIAT* a contender as a possible therapeutic target, as well as a diagnostic tool [58]. 

### 4.3. DSCAM Antisense RNA 1 (DSCAM-AS1)

*DSCAM-AS1* is implicated in multiple tumorigenic processes, from DNA replication to chromosome separation, and is highly expressed in breast cancer and is regulated by estrogen; furthermore, it associates with clinical aggressiveness in breast cancer and also associates with estrogen as well as tamoxifen-associated gene signatures [43]. Miano et al. have found that *DSCAM-AS1* belongs to a group of transcripts regulated by unliganded ERα binding to the genome (Apo-ERα) [59]. Typically, ERα is activated by estrogen leading to purely luminal breast cancer, but it has also been found to function in a hormone-independent manner leading to activation of a more aggressive basal type cancer [59]. The mechanism by which *DSCAM-AS1* provides specific RNA-binding protein stability is still being studied, but so far has identified preferential binding to hnRNPL, a nuclear protein known to stabilize mRNA, among its other roles [60]. Niknafs et al. further confirmed that *DSCAM-AS1* expression is induced by estrogen upon ER binding to the *DSCAM-AS1* promoter and showed how the interaction between *DSCAM-AS1* and hnRNPL leads to the more aggressive cancer phenotype. They found that knockdown of *DSCAM-AS1* by shRNA reduced the proliferative ability of ER+ cell lines, diminished cell invasion capabilities, and limited cell colony formation. When overexpressed in T47D, ZR75-1, and MDAMB231 cells, an increase in cellular invasion was observed. Also, as observed in xenograft assays, tumor growth was attenuated in the absence of *DSCAM1*. Further, it was found that tamoxifen resistance is associated with higher *DSCAM-AS1* expression, along with higher ERα, likely due to the continued absence of estrogen that leads to a compensatory signaling pathway. Additionally, the study found that knocking down both *DSCAM-AS1* and hnRNPL in tamoxifen-resistant ER+ cells leads to a reduction in cell proliferation [43]. This interaction could be an indicator of its therapeutic value in combating tamoxifen resistance in ER+ breast cancer, and requires further investigation. 

### 4.4. Long Intergenic Non-Protein Coding RNA 472 (LINC00472) and Long Intergenic Non-Protein Coding RNA (LINC01016)

*LINC00472* has been associated with lung adenocarcinoma, epithelial ovarian cancer, and breast cancer cell lines [44]. Genomic data indicates that the *LINC00472* promoter has an ERα binding site, is upregulated by ERα, and when overexpressed, *LINC00472* interacts with “nuclear factor kappa-light-chain-enhancer of activated B cells (NF-kB)” transcription factor [61], a known promoter of oncogenic expression [61]. *LINC00472* inhibits NF-kB signaling by repressing the phosphorylation of p65 and IkBα, resulting in a blocked transduction pathway and no tumor progression, both in vivo and in vitro [61]. Wang et al. also observed increased *LINC00472* expression in ER+ tumors, and its inhibition of pathway-activated tumor growth correlates to less tumor growth and improved patient outcome. 

Another long intergenic non-coding RNA, *LINC01016*, has been shown to be directly regulated by ERα, based on binding site overlap with regions that are commonly associated with open chromatin, with ERα binding to *cis*-regulatory regions. It is also rapidly induced by estrogen treatment, and ER+ tumors show high expression levels of *LINC01016* [45]. While a more detailed assessment is necessary, Jonsson et al. found that the patients with higher *LINC01016* expression had better clinical outcomes, so it is possible that *LINC01016* could be a new significant survival biomarker. Altogether, *LINC00472* and *LINC01016* plausibly regulate estrogen-dependent signaling with better clinical outcomes, indicative of tumor suppressor function in ER+ breast cancer. 

### 4.5. Eosinophil Granule Ontogeny Transcript (EGOT)

*EGOT* is an antisense intronic lncRNA whose reduced expression is associated with larger tumor size and increased lymph node metastasis, with a clear, direct link to less aggressive and higher survival of breast cancer patients [62]. High *EGOT* expression correlates with longer disease-free and overall survival in viral infections, blood cancers, glioma, and breast cancers, but not in gastric cancers since high expression correlates with shorter survival times [63]. Xu et al. found that estrogen binding reduced the expression of *EGOT* in a dose-dependent manner, and could be a predictive marker of paclitaxel sensitivity due to the link between paclitaxel sensitivity and abnormal autophagy when *EGOT* is highly expressed [48]. Autophagy induction is the mechanism targeted by paclitaxel, a microtubule-disrupting drug that reduces autophagy initiating genes, thereby providing an estimation of the effectiveness of paclitaxel [48,64]. *EGOT* is a unique estrogen-regulated lncRNA, which should be explored in treating ER+ breast cancers, along with other chemotherapeutic agents.

### 4.6. Long Intergenic Non-Protein Coding RNA, Regulator of Reprogramming (LINC-ROR), and LncRNA In Non-Homologous End Joining (NHEJ) Pathway 1 (LINP1)

*LINC-ROR* is a recently studied lncRNA that has been reported to be highly expressed in triple-negative breast cancer. *LINC-ROR* was upregulated when cells were deprived of estrogen leading to estrogen-independent growth, as well as tamoxifen resistance of breast cancer cells [49]. This was shown by performing cell proliferation and colony formation assays in CRISPR/Cas9-mediated *LINC-ROR* knockout MCF-7 cells. Similarly, a recent study by Ma T et al. suggests a lncRNA named *LINP1*, which is repressed by estrogen treatment, may also play a role in tamoxifen resistance in breast cancer cells [50]. The expression of *LINP1* was shown to be upregulated in tamoxifen-resistant breast cancer cells, and its subsequent knockdown using targeted siRNAs resulted in increased tamoxifen-induced apoptosis, and thus less resistance to tamoxifen treatment. Under normal circumstances, *LINP1* transcription is repressed in the presence of estrogen, but when treated with estrogen antagonist tamoxifen, the repression of *LINP1* transcription is alleviated. In the absence of *LINP1* inhibition, there was an associated decrease in the ERα receptor protein level and thus estrogen activity, potentially explaining how the lncRNA controls the tamoxifen resistance [50]. These lncRNAs could play key roles as diagnostic and therapeutic targets in hormone-refractory breast cancer. 

## 5. Conclusions

While once thought to be an unexciting part of the genome, the new annotations of lncRNAs and eRNAs are merely scratching the surface of the critical roles played by a majority of the eukaryotic genome in regulating various pathways such as estrogen signaling. ERα and its associated coregulators constitute the regulatory complex that is involved in estrogen-dependent signaling, and identification of estrogen-regulated ncRNAs may reveal new intricate details by which estrogen drives cellular processes. The wide variety of cell-specific non-coding RNAs with unknown function imply the vast majority remain unannotated and yet to be characterized. The role hormones play in male and female physiology at all ages is significant, and ncRNAs, in combination with estrogen, appear to be catalysts in multiple mechanisms ranging from carbohydrate metabolism to genomic signaling and epigenetic mechanisms [65]. Another function of ncRNAs that has yet to be explored is their plausible interaction with environmental factors, which are an essential part of estrogen-dependent physiology [66]. Interrogating these complex interactions in a cell- and tissue-specific manner may reveal the mechanistic details that are hardly known with respect to estrogen-mediated physiology in both normal and pathological conditions. Collectively, these reports suggest that detailed genetic analyses of estrogen-regulated ncRNAs must continue to understand the dynamics of estrogen-dependent signaling.

## Figures and Tables

**Table 1:** Estrogen-regulated eRNAs.

eRNA	Regulation by E2	Function
*TFF1e*	Induced	Enhancer: promoter looping [29]
*FOXC1e*	Induced	Enhancer: promoter looping [29]
*CA12e*	Induced	Enhancer: promoter looping [29]

**Table 2:** Estrogen-regulated lncRNAs.

LncRNA	Regulation by E2	Function
*LncRNA152*	Induced	Proliferation [35]
*LncRNA67*	Induced	Proliferation [35]
*H19*	Induced	DNA methylation [38]
*HOTAIR*	Induced	Transcriptional regulation [41]
*MIAT*	Induced	Cell cycle progression [42]
*DSCAM-AS1*	Induced	Cell cycle progression [43]
*LINC00472*	Induced	Tumor suppressor [44]
*LINC01016*	Induced	N/A [45]
*MALAT1*	Repressed	Transcriptional regulation [46,47]
*EGOT*	Repressed	Autophagy pathway [48]
*LINC-ROR*	Repressed	Silences ER-signaling [49]
*LINP1*	Repressed	G1-phase cell cycle arrest, apoptosis [50]

## Abbreviations

mRNA	Messenger RNA
rRNA	Ribosomal RNA
tRNA	Transfer RNA
GRO-seq	Global nuclear Run-On Sequencing
PRO-seq	Precision nuclear Run-on Sequencing
ChIP-seq	Chromatin Immunoprecipitation Sequencing
ncRNA	Non-coding RNA
LncRNA	Long Non-coding RNA
eRNA	Enhancer RNA
ERα	Estrogen Receptor α
T-UCRs	Transcribed Ultra-Conserved Regions
ER+	ER-positive
*LncRNA152*	Long Non-coding RNA 152
*LncRNA 67*	Long Non-coding RNA 67
*H19*	H19 Imprinted Maternally Expressed Transcript
ceRNA	Competing Endogenous RNA
*HOTAIR*	HOX Transcript Antisense RNA
H3K27me3	H3 lysine 27 trimethylation
PRC2	Polycomb Repressive Complex 2
PRC1	Polycomb Repressive Complex 1
*MIAT*	Myocardial Infarction-Associated Transcript
*DSCAM-AS1*	DSCAM Antisense RNA 1
NF-kB	Nuclear Factor kappa-light-chain-enhancer of activated B cells
*LINC00472*	Long Intergenic Non-Protein Coding RNA 472
*LINC01016*	Long Intergenic Non-Protein Coding RNA 1016
*MALAT1*	Metastasis-Associated Lung Adenocarcinoma Transcript 1
*EGOT*	Eosinophil Granule Ontogeny Transcript
*LINC-ROR*	Long Intergenic Non-Protein Coding RNA, Regulator of Reprogramming
*LINP1*	LncRNA In Non-homologous End Joining (NHEJ) Pathway 1
